# Characterization of cyclitic membranes by ultrabiomicroscopy in patients with pars planitis

**DOI:** 10.1186/s12348-020-0194-7

**Published:** 2020-01-27

**Authors:** Luz Elena Concha del Río, Gonzalo Alejandro Duarte González, Mariana Mayorquín Ruiz, Lourdes Arellanes-García

**Affiliations:** 1 Inflammatory Eye Diseases Clinic, Hospital Dr. Luis Sánchez Bulnes, Asociación para Evitar la Ceguera en México (APEC), Vicente García Torres 46, Col. Barrio San Lucas, Coyoacán, Mexico City, Mexico; 2 Ultrasound Service, Hospital Dr. Luis Sánchez Bulnes. Asociación para Evitar la Ceguera en México (APEC), Vicente García Torres 46, Col. Barrio San Lucas, Coyoacán, Mexico City, Mexico

**Keywords:** Pars planitis, Ultrasound biomicroscopy, Cyclitic membrane, Pars plana, Uveitis

## Abstract

**Background:**

In previous studies, authors use ultrasound biomicroscopy (UBM) to analyze the characteristics of cyclitic membranes and the associated complications in patients with pars planitis. However, there are no reports regarding the prevalence of cyclitic membranes or complications at diagnosis and during follow-up.

**Purpose:**

To describe the characteristics and complications of cyclitic membranes, as determined by UBM in patients with pars planitis using AVISO-S™ (Quantel Medical) equipment with a 50-MHz linear probe with a focus at the pars plana.

**Design:**

This retrospective study reviewed UBM images of patients diagnosed with pars planitis, from the Inflammatory Eye Disease Clinic in Mexico City from January 2010 to June 2016.

**Results:**

Cyclitic membranes were observed in the first UBM image in 67 eyes (56.7%) and during follow-up in 81 eyes (68.62%). In 67 eyes (82.71%), the cyclitic membranes extended through one or two quadrants. Extension toward the posterior lens capsule was recognized in 15 eyes (18.52%) and extension toward the peripheral retina in 12 eyes (14.81%). Complications included ciliary body detachments in 10 eyes (12.35%) and peripheral retinal traction in 8 eyes (9.88%).

**Conclusions:**

UBM is a valuable tool for the diagnosis of cyclitic membranes at admittance and during follow-up of patients with pars planitis; it helps the clinician to detect this complication early.

## Introduction

Pars planitis (PP) is a chronic inflammatory ocular disease characterized by lymphocytic infiltration in the pars plana and vitreous cavity, as well as the presence of peripheral retinal vasculitis [1]. The SUN working group reserves the term PP for a subtype of intermediate uveitis associated with the formation of fibrovascular tissue, aggregation of inflammatory cells at the pars plana (“snowbanks”), and accumulations of inflammatory cells in the vitreous cavity (“snowballs”) in at least one eye and in the absence of an associated systemic disease [2]. Usually, this condition is bilateral, although it may manifest asymmetrically [1, 3].

PP has a bimodal form of presentation, with an increased frequency in children and young adults [4]. Previous studies have reported its incidence to be up to 1.5 cases per 100,000 habitants per year [5]. At ocular inflammatory disease clinics in third-level reference centers, the incidence is reported to range from 2.4 to 15.4% [6]. In Mexico, the incidence of this pathology in a tertiary referral center during a 9-year study period was 2.92% among all uveitis patients [1].

The presence of cyclitic membranes (CM) has been reported in up to 15.3% of eyes with PP that were clinically examined by slit lamp or indirect ophthalmoscopy [1]. Although CM are not the most frequently observed complication of PP, they become a matter of concern when present; their presence may worsen the final visual prognosis. CMs are fibrovascular proliferations that extend from the inner side of the ciliary body to the vitreous base and the peripheral retina [7], spreading along in the space between the posterior lens capsule and the anterior hyaloid. They are formed by the migration of macrophages through the ciliary epithelium, which then differentiate into fibroblasts or other connective tissue cells, generating a fibrous tissue with contractile properties [7]. When cataract or retina surgery is performed, CMs are a key factor for deciding the surgical technique and intraocular lens implantation. When there is a ciliary body detachment, phthisis bulbi is more frequent. CM have been reported in up to 15.3% of eyes with PP that were clinically examined by slit lamp or indirect ophthalmoscopy [1]. Contraction of CM can produce severe complications such as ciliary body detachment, choroidal detachments, and peripheral retinal detachments. Ciliary body detachment causes a decrease in the production of aqueous humor, which leads to ocular hypotension, hypotensive maculopathy, and, finally, phthisis bulbi [7].

Clinical examination of the ciliary body and pars plana is difficult in patients with PP because of the media opacity and poor pupillary dilatation due to posterior synechia, which was present in most of our patients at diagnosis. Complementary examinations, such as those using anterior segment optical coherence tomography (AS-OCT) and ultrasound biomicroscopy (UBM), are useful for imaging the anterior segment. The main limitation of AS-OCT is the optical principle that makes it suitable only for imaging through clear media and with a penetration depth of 6 mm only. In contrast, UBM allows for high-resolution images at the expense of low-signal tissue penetration, allowing cross-sections of the anterior segments and other deeper structures like the lens, zonule, ciliary body, pars plana, peripheral retina, and anterior vitreous cavity [8, 9]. The utility of UBM for intermediate uveitis patients was first described by García-Feijoo et al [10]. The aim of this study is to report the presence of CM using UBM and to describe their characteristics and associated complications in PP patients.

## Methods

This retrospective study reviewed UBM images in the medical records of patients diagnosed with PP from the Ocular Inflammatory Disease Clinic at the Asociación Para Evitar la Ceguera Hospital “Dr. Luis Sánchez Bulnes” in Mexico City from January 2010 to June 2016.

A diagnosis of pars planitis was made based on the SUN classification [2] and confirmed using angiographic criteria: venous capillaries hyperfluorescence (“fern pattern hyperfluorescence”), optic disk hyperfluorescence, and staining of the vessel walls [1]. We included eyes examined with UBM at diagnosis and at least once during the follow-up period. Patients for whom the PP diagnosis was modified during follow-up were excluded.

The study was conducted under the tenets of the Declaration of Helsinki and approved by the hospital’s ethical and research committee.

Ocular Ultrasound Service performed the UBM examinations. AVISO-S™ (Quantel Medical) equipment was used with a 50-MHz linear probe, achieving an 11-mm penetration depth in the intraocular tissues.

A standardized protocol was followed. The patient remained in the supine position under standard illumination after the application of topical tetracaine in each eye. During each exploration, tracking was performed with 360° longitudinal scans of the eyeball, focusing on the pars plana. The following echographic variables were analyzed in each of the eyes: the presence of CM, circumferential extension of CM measured in meridians, anterior extension toward the lens and/or ciliary body, posterior extension to the pars plana, retinal traction, and the ciliary body detachment. For the circumferential extension of CM, the number of involved meridians were evaluated. A meridian is defined as one measure when dividing the eye like a 12-h clock. One-quadrant involvement was considered when it included 1–3 meridians; two-quadrant when it included 4–6 meridians; three-quadrant when it included 7–9 meridians; and four-quadrant when it included 10–12 meridians.

Information was collected in a database and analyzed using SPSS version 20 (SPSS Inc., Chicago, Illinois, USA). Descriptive statistics were applied to the results. A linear regression analysis was performed to correlate gender and the presence of CM at the beginning and end of the study. Bivariate analysis of the other variables was performed using the Chi-square test and Spearman’s rank correlation coefficient, as appropriate. *P* values < 0.05 were considered statistically significant.

## Results

We included 118 eyes from 66 patients, with a 78-month follow-up. Forty-one patients (62.12%) were male. The mean age at admission was 10.85 ± 6.03 years. Sixty-one were right eyes (51.69%). The disease was bilateral in 78.78% of the patients (Table 1).

**Table 1:** Demographic characteristics

Characteristics	Total
Number of patients/number of eyes	66/118
Gender (male/female)	41/25 (62.12%/37.88%)
Age at diagnosis	10.85 ± 6.03 years (3–36 years)
Time from PP diagnosis to first UBM	2.6 ± 7.2 months (0–36 months)
Presence of CM at presentation (UBM)	67 eyes (56.78%)
Presence of CM at last visit	81 eyes (68.64%)

At the time of PP diagnosis, we found CM in 67 eyes (56.78%) using UBM (Table 1). A second UBM was performed during follow-up, and 14 eyes show a CM. CM developed in an average time period of 18.86 ± 9.1 months.

**Fig. 1:**
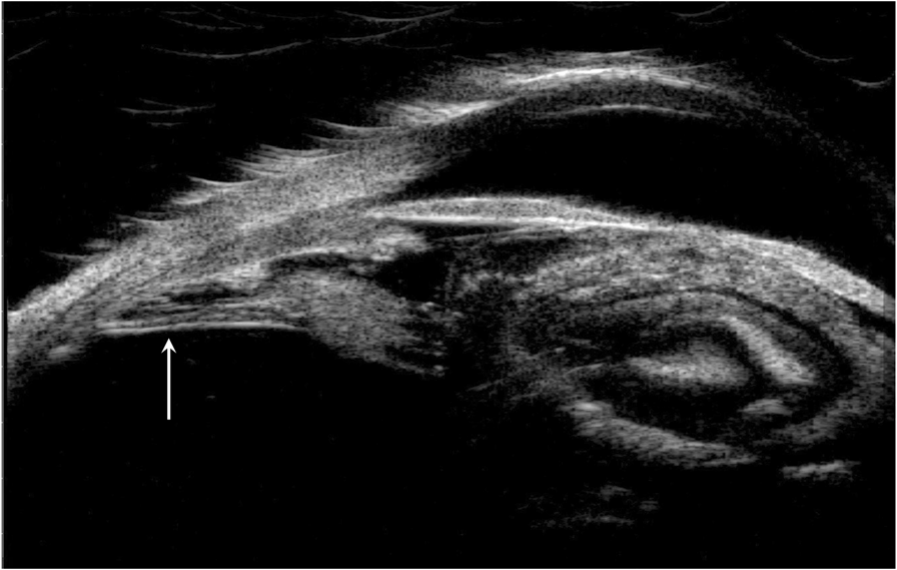
Anterior extension of cyclitic membrane towards lens and ciliary body. 1772 × 1124 mm (72 × 72 DPI)

**Fig. 2:**
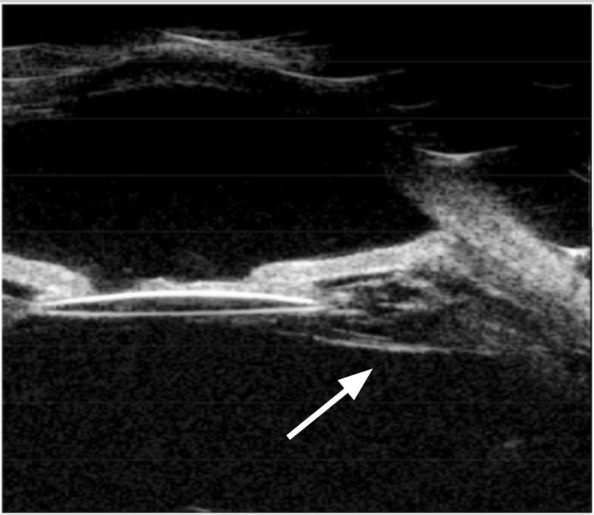
Anterior extension of cyclitic membrane towards intraocular lens. 800 × 689 mm (72 × 72 DPI)

**Fig. 3:**
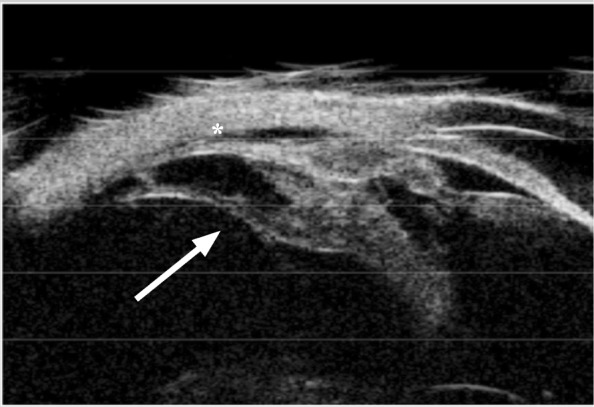
Posterior extension of cyclitic membrane to pars plana (arrow) with peripheral choroidal detachment (asterisk). 1585 × 964 mm (72 × 72 DPI)

At the end of the study, we observed CM in 81 eyes (68.64%). None decreased in size. Quadrant extension is shown in Table 2. Anterior extension toward the posterior lens capsule was recognized in 15 eyes (18.52%), as shown in Figs. 1 and 2. Twelve eyes with CM (14.81%) had posterior extension toward the peripheral retina, as shown in Figure 3. Complications included ciliary body detachments in 10 eyes (12.35%); none of these eyes had hypotony or evolved to phthisis; 8 eyes (9.88%) had peripheral retinal traction with ciliary body detachment; none required surgical treatment during follow-up.

**Table 2:** UBM characteristics of cyclitic membranes and complications

UBM characteristics		Total
Circumferential extension of CM	1 quadrant	37 eyes (45.68%)
	2 quadrants	30 eyes (37.03%)
	3 quadrants	4 eyes (4.94%)
	4 quadrants	10 eyes (12.35%)
Anterior extension of CM		15 eyes (18.52%)
Posterior extension of CM		12 eyes (14.81%)
Ciliary body detachment		10 eyes (12.35%)
Retinal traction		8 eyes (9.88%)

There was no significant correlation between the age at diagnosis and the presence of CM at the beginning and end of the study period. There was also no correlation between these two variables and patients’ gender. Also, patient age at diagnosis and gender did not show correlations with the number of involved quadrants. A statistically significant association between the male gender and the risk of developing CM at the end of the study was found (OR 2.6, 95% CI 1.1–5.8) (*P* = 0.018). There was no correlation between the presence or development of CM and the given treatment; all patients were treated with topical prednisolone, oral prednisone, and methotrexate.

## Discussion

UBM is a non-invasive eye examination method that visualize structures such as the ciliary body and peripheral retina. In PP patients, these structures may be difficult to evaluate in routine ophthalmological examinations due to the poor dilatation and media opacities commonly found in these cases [11].

CM develop in the inner surface of the ciliary body in chronic inflammatory processes [12]. They stretch across the back of the lens, anterior hyaloid surface, lens implants or pupil in aphakic eyes [1]. UBM allows us to examine the presence, localization, and extension of CM in PP patients.

Of the studied eyes, 68% presented CM detected by UBM. This percentage is higher than the 15.3% reported by us in a previous study, in which CM was diagnosed via clinical examination [1]. In 67 eyes (56.78%), CM was observed at the time of PP diagnosis. Moreover, in most cases (82.71%), CM involved one or two quadrants, mainly inferior ones.

UBM helps practitioners make decisions regarding treatment. For example, it may help determine the optimal puncture site for intravitreal injections, avoiding areas where CM traction can be generated. In patients requiring vitreo-retinal surgery, UBM helps us determine the entry-port location; in patients with vitreoretinal traction, it may help us recognize the risk of retinal detachment [11].

Considering that almost 1 in 5 affected eyes in our study presented CM extension to the posterior lens capsule, UBM helps in weighting the risks and benefits of implanting a lens in the capsular bags in these cases. Over time, CM could be developed with an extension of at least one quadrant; implantation of an intraocular lens could lead to a predisposition to lens tilting, angle closure, and/or anterior-chamber inflammation. Of the eyes included in our study, 12% presented ciliary body detachment. These eyes have a higher risk of ocular hypotension, maculopathy, and, eventually, phthisis bulbi [9]. In these cases, early lensectomy may prevent chronic hypotony. Roters et al. reported that in eyes with chronic inflammation, CM obstruct aqueous production and that, after their removal, it is rare for intraocular pressure to return to normal levels [13].

Ciliary processes have been described as present in patients with membranes adhered to the ciliary body [9], but the anatomy has not yet been described. In our study, we did not find changes in the morphology of the ciliary processes.

The main limitation of this study was its retrospective nature, which lead to missing data such as the clinical diagnosis of CM, correlation with visual acuity, and long-term follow-up. Despite these limitations, this study has some strengths: standardized inclusion criteria, strict diagnostic criteria, and an ample cohort of patients.

Within the group of patients who did not present CM at the initial UBM examination, and develop CM in the follow-up, a new line of investigation was opened to determine if the severity of anterior chamber and/or vitreous cavity inflammation and the type of treatment is associated with a higher risk of developing this complication. This goes in line with what Lamb described [12], where the vitreous adjacent to the ciliary body is the earliest stage in the development of the cyclitic membrane and in pars planitis the vitreous is the main site of inflammation [2]. In this descriptive study, no risk factor analysis was performed.

## Conclusion

In summary, UBM is an important tool to detect CM in patients with PP, and it plays a role in the early detection of sight-threatening complications. The percentage of CM detected by UBM is much higher than the detected during clinical eye examinations, which demonstrates the contribution of this study. Our findings have opened a new line of investigation to determine which factors are associated with the development and growth of CM and their effects on visual prognosis.

## Data Availability

The datasets used and/or analyzed during the current study are available from the corresponding author on reasonable request.

## Abbreviations

AS-OCT: Anterior Segment Optical Coherence Tomography
CM: Ciclytic membrane
PP: Pars planitis
UBM: Ultrasound biomicroscopy
